# Estimating the Transmission Kernel for Lumpy Skin Disease Virus from Data on Outbreaks in Thailand in 2021

**DOI:** 10.3390/v15112196

**Published:** 2023-10-31

**Authors:** Veerasak Punyapornwithaya, Roderick Salvador, Wittawat Modethed, Orapun Arjkumpa, Chaiwat Jarassaeng, Georgina Limon, Simon Gubbins

**Affiliations:** 1 Research Center of Veterinary Biosciences and Veterinary Public Health, Chiang Mai University, Chiang Mai 50100, Thailand; veerasak.p@cmu.ac.th; 2 Faculty of Veterinary Medicine, Chiang Mai University, Chiang Mai 50100, Thailand; wittawat_m@cmu.ac.th; 3 College of Veterinary Science and Medicine, Central Luzon State University, Science City of Muñoz, Nueva Ecija 3120, Philippines; roderick.salvador@clsu2.edu.ph; 4 Animal Health Section, The 4th Regional Livestock Office, Department of Livestock Development, Khon Kaen 40260, Thailand; arjkumpa@hotmail.com; 5 Faculty of Veterinary Medicine, Khon Kaen University, Khon Kaen 40002, Thailand; chajar@kku.ac.th; 6 The Pirbright Institute, Pirbright, Surrey GU24 0NF, UK; georgina.limon-vega@pirbright.ac.uk

**Keywords:** lumpy skin disease, kernel transmission, cattle, epidemiology, Thailand

## Abstract

Nationwide outbreaks of lumpy skin disease (LSD) were observed in Thailand in 2021. A better understanding of its disease transmission is crucial. This study utilized a kernel-based approach to characterize the transmission of LSD between cattle herds. Outbreak data from the Khon Kaen and Lamphun provinces in Thailand were used to estimate transmission kernels for each province. The results showed that the majority of herd-to-herd transmission occurs over short distances. For Khon Kaen, the median transmission distance from the donor herd was estimated to be between 0.3 and 0.8 km, while for Lamphun, it ranged from 0.2 to 0.6 km. The results imply the critical role that insects may play as vectors in the transmission of LSD within the two study areas. This is the first study to estimate transmission kernels from data on LSD outbreaks in Thailand. The findings from this study offer valuable insights into the spatial transmission of this disease, which will be useful in developing prevention and control strategies.

## 1. Introduction

Lumpy skin disease (LSD) is a viral disease that primarily affects cattle, causing significant economic losses to the livestock industry worldwide [1,2,3]. The disease is caused by lumpy skin disease virus (LSDV), a member of the *Capripoxvirus* genus within the *Poxviridae* family [4]. Clinical signs of LSD include the appearance of nodules on the skin; fever; reduced milk production; weight loss; and, in severe cases, death [5,6]. The morbidity rate is 3–85%, depending on the animal immune status and the abundance of insect vectors, while the mortality rate is usually less than 5% [1,7]. Biting insects such as mosquitoes, ticks, and flies act as mechanical vectors for LSDV transmission [3,8,9]. Direct contact between infected and healthy animals and exposure to contaminated equipment or feed can also contribute to the spread of LSD [8,10]. At the farm level, LSD has an economic impact through a decline in milk production, weight loss, inferior hide quality, abortion, and mortality [7,11,12,13]. The World Organization for Animal Health (WOAH) has listed LSD as one of the most important transboundary diseases [2,14].

Since 2012, the geographical range of LSD has expanded from Africa to other regions of the world, including Europe, the Middle East, and Asia [15,16]. In recent years, LSD outbreaks have been reported in numerous countries in Asia [15,16,17,18,19,20]. It is estimated that LSD in South, East, and Southeast Asian countries causes 1.45 billion dollars in direct losses through its impact on livestock production [21]. Among Asian countries, Thailand reported the highest number of LSD outbreaks in 2021, according to World Animal Health Information System (WAHIS) data [15]. Thailand reported its first LSD outbreak in a beef cattle herd in March of 2021 [22]. Thereafter, multiple outbreaks were reported in cattle across Thailand [23]. The large number of outbreaks led to substantial economic losses and had a negative impact on farmers’ livelihoods. Because the cattle industry in Thailand plays a crucial role in the national economy and in food security, effective prevention and control of LSD is a high priority for the country [23].

Understanding the transmission dynamics of LSD is essential for the development of targeted interventions and the efficient allocation of resources. One useful tool for helping to understand spatial patterns of spread is the transmission kernel, which describes how the probability of transmission between epidemiological units depends on the distance between them. This approach can provide valuable insight into patterns of disease spread and the potential impact of control measures [24,25,26,27].

The aim of this study was to investigate the role of distance in LSDV transmission among herds in two cattle farming areas in Thailand using a kernel-based approach. By examining the transmission patterns of LSDV in these areas, this research will offer valuable insights into the epidemiology of LSD. These can then be used to inform targeted control strategies, such as vaccination campaigns, vector control measures, or movement restrictions, in specific provinces.

## 2. Materials and Methods

### Data

**Figure 1:**
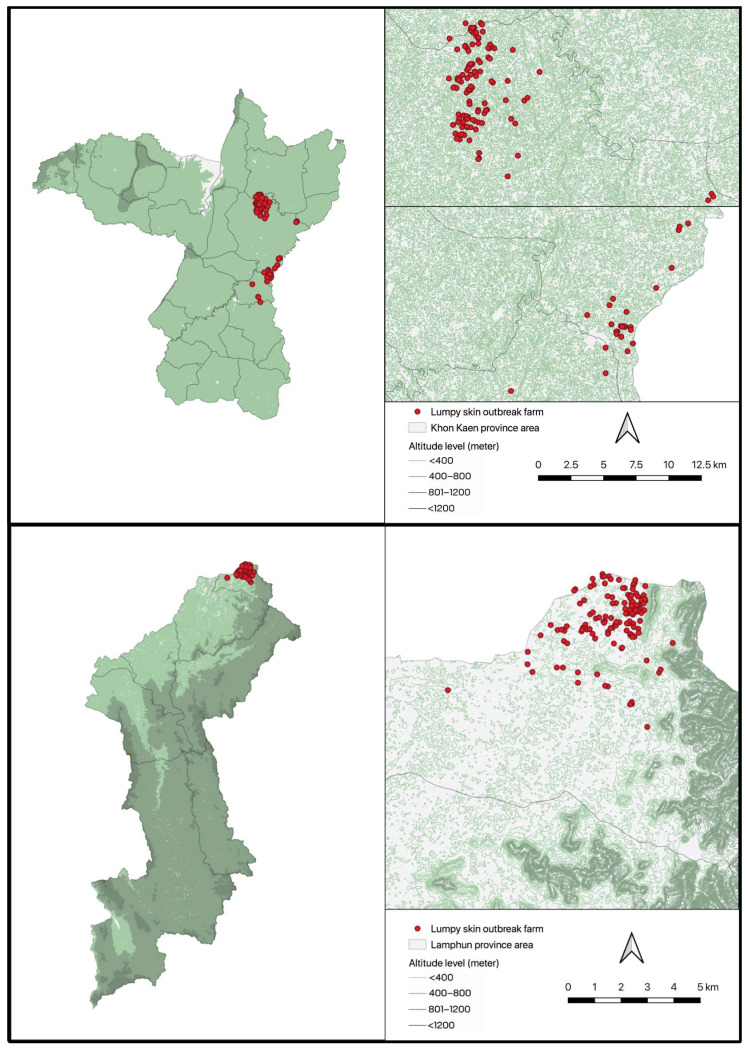
Maps depicting the locations of lumpy skin disease outbreaks in the Khon Kaen (**upper panels**) and Lamphun (**lower panels**) provinces, Thailand. Each dot represents the location of a herd.

This study used data on LSDV outbreaks in the Khon Kaen and Lamphun provinces (Figure 1). These outbreaks occurred between 5 May and 8 July 2021 in Khon Kaen, and between 5 June and 27 August 2021 in Lamphun. Outbreaks were identified through a combination of farmers reporting suspect cases and veterinary authorities surveying cattle farms in areas experiencing outbreaks. An outbreak was defined as a farm having at least one LSD case. An LSD case was defined as individual bovine exhibiting clinical signs of LSD, specifically elevated, round, and solid nodules ranging between 1 and 7 cm in diameter [22]. No vaccination was conducted in these provinces either before or during the outbreaks.

**Figure 2:**
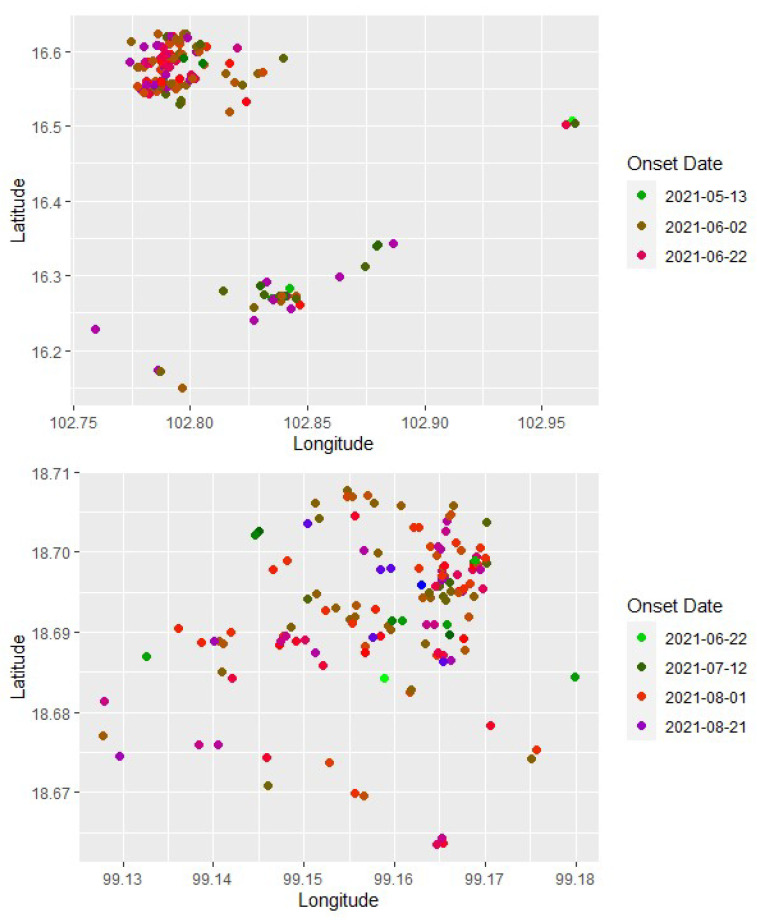
The geographical location of lumpy skin disease outbreaks in the Khon Kaen (**upper panel**) and Lamphun (**lower panel**) provinces, Thailand. Each dot represents a cattle herd, and the color of the dot indicates the onset date of the outbreak (indicated to the right of each panel).2.2. Modeling Approach.

The maps depicting outbreak locations in Khon Kaen and Lamphun provinces (Figure 1) were created using QGIS (https://www.qgis.org; accessed on 1 September 2023) and publicly available shape files (https:/www.earthdata.nasa.gov; accessed on 1 September 2023). The geographical coordinates of each herd and its onset date in Khon Kaen and Lamphun (Figure 2) were visualized using R (https://www.r-project.org; accessed on 1 September 2023).

The transmission kernel, *K*(*d*), can be interpreted as one minus the cumulative distribution function for the distances, *d*, between donor and recipient herds. Accordingly, the kernel can be estimated using a likelihood given by

(1) $$L=\prod_{x=1}^{x_{\max}}{\left(K\left(x+0.5\right)-K\left(x-0.5\right)\right)}^{N_{x}},$$

where *N_x_* is the number of transmission events where the distance between donor and recipient herds is between *x* − 0.5 and *x* + 0.5 km.

Four functional forms for the transmission kernel, *K*(*d*), were explored, which differed in their shapes and in the proportion of transmission that occurred at longer distances. The first (fat-tailed) is given by

(2) $$K\left(d\right)={\left(1+{\left(\frac{d}{d_{0}}\right)}^{2}\right)}^{-1},$$

where *d*_0_ is the median transmission distance. The second (Gaussian) and third (exponential) are given by

(3) $$K\left(d\right)=\exp\left(-{\left(\alpha d\right)}^{2}\right)$$

and

(4) $$K\left(d\right)=\exp\left(-\alpha d\right),$$

respectively, where *α* is the reciprocal of the mean transmission distance. The fourth (alternative fat-tailed) is given by

(5) $$K\left(d\right)={\left(1+\frac{d}{d_{0}}\right)}^{-\gamma },$$

where *d*_0_ is the distance scaling and *γ* is the kernel parameter (which controls how rapidly the kernel declines with distance).

The parameters of the four transmission kernels, (2)–(5), were estimated using maximum likelihood methods, and the fits of the different kernels were compared using the Akaike information criterion (AIC) [28]. Separate kernels were estimated for the Khon Kaen and Lamphun provinces.

For each outbreak, the donor herd (i.e., the source of infection for the outbreak) was assumed to be that which experienced the nearest outbreak that had an onset date before that of the recipient herd. Because cattle in the donor herd would be showing clinical signs of LSD (by the case definition; see above), the herd would potentially be infectious to other herds. Furthermore, the time scales of the outbreaks in both provinces were short enough that recovery from infection in the donor herds could reasonably be neglected.

To test the sensitivity of parameter estimates and model fit according to the assumption that the nearest outbreak was the source of infection for a herd, the donor herds were reassigned for a proportion of recipient herds by selecting another outbreak with an onset date prior to that of the recipient at random, regardless of the distance between the donor and the recipient. This reassignment was repeated 1000 times, and parameters were estimated for each reassignment.

## 3. Results

**Figure 3:**
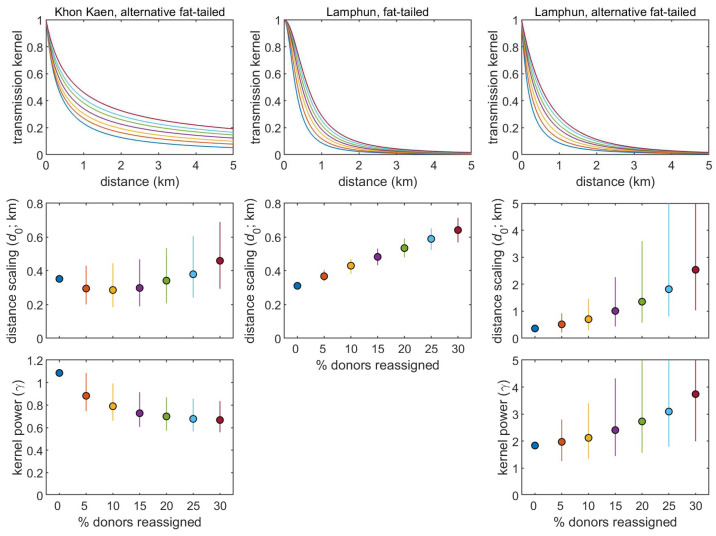
Transmission kernels estimated from data on outbreaks of lumpy skin disease virus in the Khon Kaen and Lamphun provinces, Thailand. The plots show the sensitivity of the estimated kernels to the assumption that each herd acquired LSDV from the nearest infected herd. This was assessed by reassigning the donor for a proportion of recipient herds to any infected herd. Results are shown for 1000 reassignments of donor–recipient pairs, with circles and error bars showing the median and 95% range for the estimates. The colors of the lines and symbols indicate the proportion of donors which were reassigned.

When estimating the transmission kernel for outbreaks in Khon Kaen province, there was a marked preference for the alternative fat-tailed kernel (5) compared with the fat-tailed (2), Gaussian (3), or exponential (4) kernels, as judged by the AIC. Furthermore, this was robust to assumptions regarding the assignment of donors (fat-tailed, ΔAIC > 13; Gaussian, ΔAIC > 592; exponential, ΔAIC > 103). In addition, the estimate for the distance scaling (*d*_0_) was robust to this reassignment, remaining around 0.3–0.4 km (Figure 3). By contrast, the estimate for the kernel power (*γ*) declined as the proportion of reassigned donors increased, resulting in much fatter tails for the kernel (Figure 3).

There was less evidence for preferring the fat-tailed kernel, (2), over the alternative fat-tailed kernel, (5), when estimating the transmission kernel for outbreaks in Lamphun province (ΔAIC < 3). However, there was a preference for either compared with the Gaussian kernel (ΔAIC > 67) and the exponential kernel (ΔAIC > 7; except when 25–30% of donors were reassigned; when the fit of all three kernels is comparable, ΔAIC < 3). The lack of a strong preference for the fat-tailed and alternative fat-tailed kernels is reflected in the similarity of the fitted kernels (Figure 3). The estimates for distance scaling (*d*_0_) in Equation (2) increased as the proportion of reassigned donors increased (Figure 3). Similarly, estimates for both the distance scaling (*d*_0_) and the kernel power (*γ*) in Equation (5) increased as the proportion of reassigned donors increased (Figure 3).

The estimated kernels for both provinces suggest that most transmission between herds occurs over short distances (Figure 3). For Khon Kaen, the median transmission distance from the donor herd was estimated to be 0.3–0.8 km, while for Lamphun, it was estimated to be 0.2–0.6 km. However, the kernel for Lamphun declined much faster with distance than that for Khon Kaen: for, Khon Kaen 95% of transmission was estimated to occur within 5.2 to 40.4 km of the donor herd, while for Lamphun, 95% of transmission was estimated to occur within 1.4–3.1 km of the donor herd (Figure 3).

## 4. Discussion

The findings from this research indicate that the spread of LSD between herds mainly occurs over short distances. Specifically, the typical transmission distance in Khon Kaen varied between 0.3 and 0.8 km, while in Lamphun, it ranged from 0.2 to 0.6 km. These observations are consistent with previous epidemiological studies concerning LSD outbreaks in Thailand. Using a space–time permutation (STP) model for analysis, seven spatio-temporal clusters were identified in Roi Et province in northeast Thailand [29]. The primary cluster had a radius of 1.8 km, and out of the six secondary clusters, four had a radius of less than 2.5 km. The remaining two secondary clusters had radii of 6.6 and 10.5 km. A previous analysis of outbreaks in Khon Kaen province using an STP model identified a primary cluster with a radius of 1.59 km, while twelve of the fourteen secondary clusters had radii of less than 1 km [30]. Similarly, an analysis of outbreaks in Albania and Israel utilizing a transmission kernel also showed that most LSDV transmission took place within a short range (less than 5 km) [24,31]. Furthermore, the kernel parameters estimated for the fat-tailed kernel from outbreaks in Albania (*d*_0_ = 0.9 km) [31] and Israel (*d*_0_ = 1.05 km) [24] were similar to those estimated for outbreaks in Lamphun province (*d*_0_ = 0.31–0.64 km) in the present study.

The present study’s findings and those from Albania and Israel show that most LSDV transmission occurs over short distances [8], which is consistent with transmission via the bites of hematophagous insects. Hence, it is reasonable to presume that insect vectors significantly contribute to disease spread in Khon Kaen and Lamphun. Furthermore, animal movement restrictions have been imposed in these areas due to the nationwide enforcement of LSD prevention and control programs by livestock authorities, thereby minimizing transmission through this route [23]. Accordingly, it is essential to pay attention to the transmission of LSDV via insect vectors. Stable flies (*Stomoxys* spp.) and *Aedes* spp. mosquitoes, which are prevalent near cattle herds in Thailand, have been identified as vectors that transmit LSDV [9,32,33]. This suggests that improving insect control methods could reduce the transmission of LSDV in the area [29].

Since the transmission of LSDV between herds in the study areas can be linked mainly to insects, it is intuitive to consider the vector control as a strategy to manage an LSD outbreak, especially where vaccination has not been carried out. Such an approach was part of the control measures against LSD implemented in Israel [31]. Nonetheless, a scientific opinion produced by EFSA did not recommend the use of insecticides, noting that there was no experimental quantitative evidence demonstrating the effectiveness of vector control in combating the spread of LSD [31]. The application of insecticides could substantially decrease the number of insects that facilitate the spread of LSD, thereby lowering the risk of disease transmission. However, estimates for the basic reproduction number, *R*_0_, for LSDV [33] suggest that the vector population would need to be reduced by 60–95% to prevent outbreaks (i.e., reducing *R*_0_ below one). Achieving this reduction presents some challenges, as it would likely require wide-scale and intensive use of insecticides that may adversely affect beneficial insects integral to ecosystem function. Furthermore, the financial burden of procuring these insecticides could represent an extra cost for cattle farmers. The feasibility of integrating insecticides into an LSD control program is beyond the scope of this study. This topic, including insect vector behavior, their habitats, and the environmental conditions that influence their populations, should be covered in collaborative research involving entomologists, epidemiologists, and animal health experts.

The differences between the transmission kernels estimated for the Khon Kaen and Lamphun provinces suggest that there are additional factors influencing the spread of LSDV between herds in these provinces. Some possible reasons for these differences include differences in vector abundance and mobility, environmental factors, host population and density, and geographical factors. Moreover, differences in the effectiveness of vector control methods during the outbreak period may also account for differences in the kernels.

When interpreting the findings of this study, it is essential to note that the cattle herds in the areas under study are closely grouped at short distances from one another. Consequently, these results may not be applicable to regions with different herd densities or production systems. In addition, the number of outbreaks used in this study was considerably lower than the number used in previous research, and this should be considered as a limitation of the study. Moreover, given that LSD outbreaks have been reported across Thailand [23], it would be beneficial for future studies to explore the epidemiology of LSDV in cattle farming provinces with different geographical traits from those previously studied. Doing so will help us to expand the current understanding of LSDV spread.

This research represents the first attempt to estimate transmission kernels for LSDV from outbreak data from Thailand, and, to the best of our knowledge, for any of the LSD epidemics in Asia. Consequently, this study’s findings can serve as foundational data, contributing to a broader understanding of LSDV transmission in the region. Notably, further research on this subject from other Asian countries could pave the way to the development of guidelines or recommendations for LSD prevention and control in the region, much like the approach taken in Europe.

## 5. Conclusions

This study utilized data from LSD outbreaks in Thailand to estimate the transmission kernel for cattle herds affected by LSDV. It highlights that the majority of herd-to-herd transmission occurs over short distances implying that insects may play an important role in the transmission of LSDV in the outbreak areas. This study provides a significant contribution to the understanding of the spatial transmission of the disease, thereby facilitating the development of effective prevention and control strategies.

## Data Availability

The data supporting the findings of this study were obtained from the Department of Livestock Development, Ministry of Agriculture and Cooperative, Thailand. The data are not publicly accessible, and in order to obtain permission to use the data, a formal letter must be sent to the DLD authority (Email: dld@info.th).
